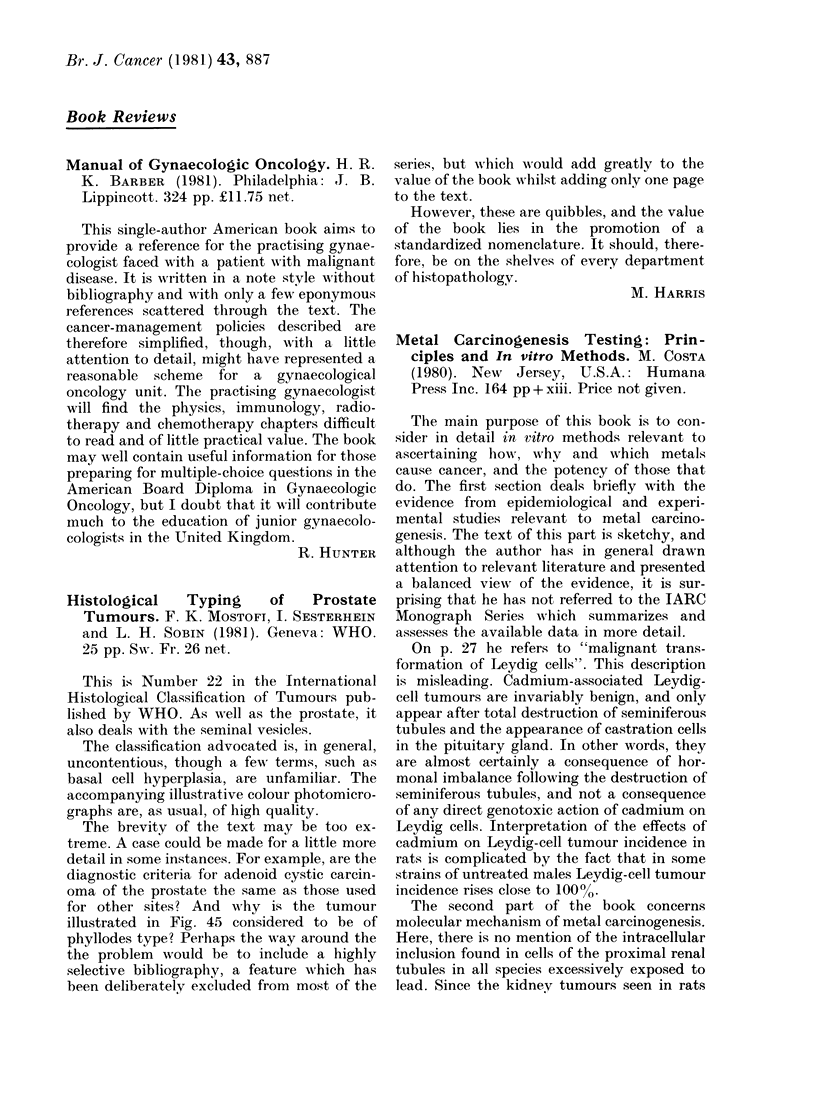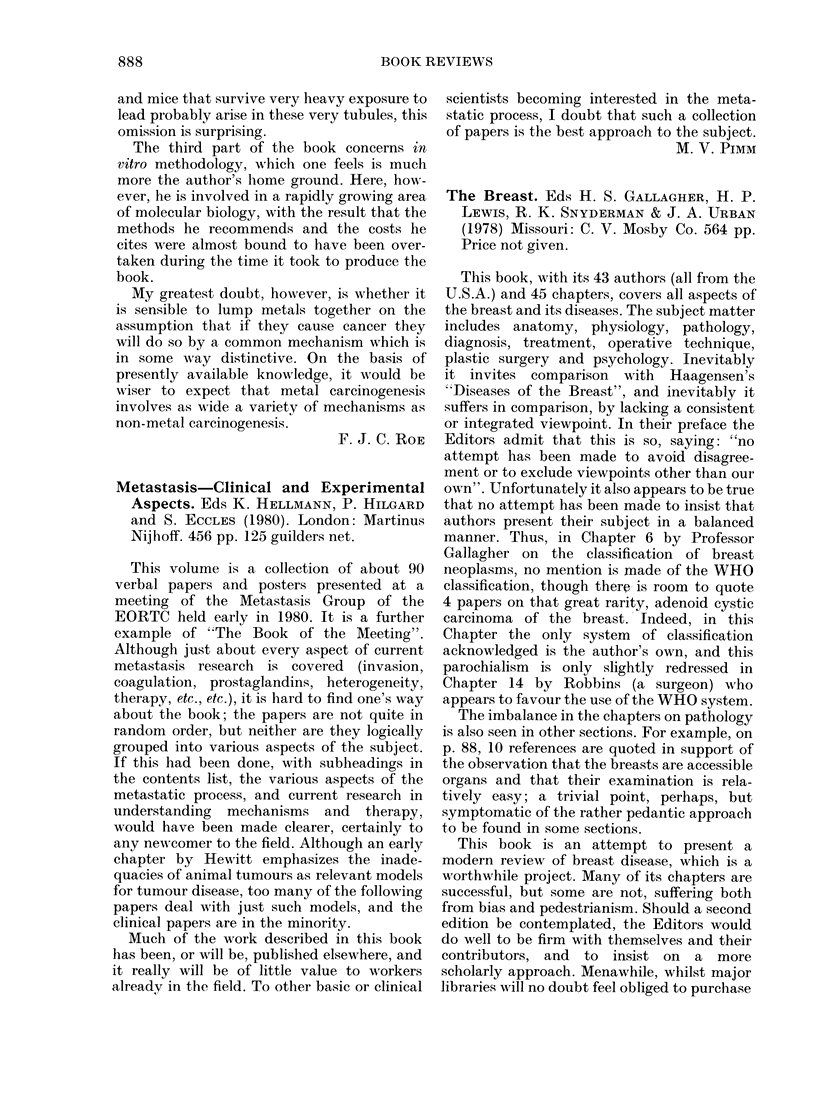# Metal Carcinogenesis Testing: Principles and In vitro Methods

**Published:** 1981-06

**Authors:** F. J. C. Roe


**Metal Carcinogenesis Testing: Principles and *In vitro* Methods.** M. Costa (1980). New Jersey, U.S.A.: Humana Press Inc. 164 pp + xiii. Price not given.

The main purpose of this book is to consider in detail *in vitro* methods relevant to ascertaining how, why and which metals cause cancer, and the potency of those that do. The first section deals briefly with the evidence from epidemiological and experimental studies relevant to metal carcinogenesis. The text of this part is sketchy, and although the author has in general drawn attention to relevant literature and presented a balanced view of the evidence, it is surprising that he has not referred to the IARC Monograph Series which summarizes and assesses the available data in more detail.

On p. 27 he refers to "malignant transformation of Leydig cells". This description is misleading. Cadmium-associated Leydig-cell tumours are invariably benign, and only appear after total destruction of seminiferous tubules and the appearance of castration cells in the pituitary gland. In other words, they are almost certainly a consequence of hormonal imbalance following the destruction of seminiferous tubules, and not a consequence of any direct genotoxic action of cadmium on Leydig cells. Interpretation of the effects of cadmium on Leydig-cell tumour incidence in rats is complicated by the fact that in some strains of untreated males Leydig-cell tumour incidence rises close to 100%.

The second part of the book concerns molecular mechanism of metal carcinogenesis. Here, there is no mention of the intracellular inclusion found in cells of the proximal renal tubules in all species excessively exposed to lead. Since the kidney tumours seen in rats and mice that survive very heavy exposure to lead probably arise in these very tubules, this omission is surprising.

The third part of the book concerns *in vitro* methodology, which one feels is much more the author's home ground. Here, however, he is involved in a rapidly growing area of molecular biology, with the result that the methods he recommends and the costs he cites were almost bound to have been overtaken during the time it took to produce the book.

My greatest doubt, however, is whether it is sensible to lump metals together on the assumption that if they cause cancer they will do so by a common mechanism which is in some way distinctive. On the basis of presently available knowledge, it would be wiser to expect that metal carcinogenesis involves as wide a variety of mechanisms as non-metal carcinogenesis.

F. J. C. Roe